# Metabolic Plasticity of Melanoma Cells and Their Crosstalk With Tumor Microenvironment

**DOI:** 10.3389/fonc.2020.00722

**Published:** 2020-05-22

**Authors:** Angelica Avagliano, Giuseppe Fiume, Alessandra Pelagalli, Gennaro Sanità, Maria Rosaria Ruocco, Stefania Montagnani, Alessandro Arcucci

**Affiliations:** ^1^Department of Public Health, University of Naples Federico II, Naples, Italy; ^2^Department of Experimental and Clinical Medicine, University “Magna Graecia” of Catanzaro, Catanzaro, Italy; ^3^Department of Advanced Biomedical Sciences, University of Naples Federico II, Naples, Italy; ^4^Institute of Biostructures and Bioimages, National Research Council, Naples, Italy; ^5^Department of Molecular Medicine and Medical Biotechnology, University of Naples Federico II, Naples, Italy

**Keywords:** cutaneous melanoma, tumor microenvironment, metabolic alterations, OXPHOS, therapeutic strategies

## Abstract

Cutaneous melanoma (CM) is a highly aggressive and drug resistant solid tumor, showing an impressive metabolic plasticity modulated by oncogenic activation. In particular, melanoma cells can generate adenosine triphosphate (ATP) during cancer progression by both cytosolic and mitochondrial compartments, although CM energetic request mostly relies on glycolysis. The upregulation of glycolysis is associated with constitutive activation of BRAF/MAPK signaling sustained by BRAF^V600E^ kinase mutant. In this scenario, the growth and progression of CM are strongly affected by melanoma metabolic changes and interplay with tumor microenvironment (TME) that sustain tumor development and immune escape. Furthermore, CM metabolic plasticity can induce a metabolic adaptive response to BRAF/MEK inhibitors (BRAFi/MEKi), associated with the shift from glycolysis toward oxidative phosphorylation (OXPHOS). Therefore, in this review article we survey the metabolic alterations and plasticity of CM, its crosstalk with TME that regulates melanoma progression, drug resistance and immunosurveillance. Finally, we describe hallmarks of melanoma therapeutic strategies targeting the shift from glycolysis toward OXPHOS.

## Introduction

Cutaneous melanoma (CM) is highly aggressive tumor characterized by an increasing worldwide incidence more distributed in the Eastern than in Western European countries (1). Among the three different CM clinically and histomorphologically steps, Vertical Growth Phase (VGP) represents the tumorigenic and /or mitogenic stage of CM (2). In VGP step, CM can metastasize to lymph nodes, brain, lung, bone, and liver albeit the size of primary tumor is still small (2). The dramatic invasive behavior of melanoma cells depends on neural crest origin of melanocytes (3). Melanoma cells derive from the malignant transformation of melanocytes affected by the constitutive activation of oncogenic signaling and cancer metabolic reprogramming, which are processes interacting each other (4–7). Oncogenic signaling pathways in malignant melanocytes can be activated by mutations in BRAF, NRAS, and neurofibromatosis type 1 (NF1) gene. Based on the genetic mutations, CM is grouped into 4 genomic subtypes represented by BRAF mutants, NRAS mutants, neurofibromatosis type 1 (NF1) mutants, and triple-wild-type tumors (8). BRAF mutations, target of therapeutic strategies, dramatically affect CM metabolism, depending mainly on glycolytic metabolism. Glycolysis leads to production of adenosine triphosphate (ATP) and building block intermediates useful for cancer progression (2, 6, 8). It is important to note that in normoxic microenvironment, melanoma cells metabolize up to 80% of glucose into lactate, and that hypoxia augments this metabolic process (6, 9, 10). This elevated rate of glucose transformation into lactate, even in normoxic microenvironment, has been showed by Otto Warburg and it is termed Warburg effect (11, 12). However, it is noteworthy that also in hypoxic melanoma microenvironment the mitochondria of cancer cells work and thus can sustain melanoma dissemination (13).

Cancer cells successfully are able to adapt to the nutritional changes and restrictions of the tumor microenvironment (TME), through dynamic modulations of both cytosolic and mitochondrial metabolic pathways in order to produce ATP during cancer progression (2). Most relevant molecular drivers that participate to melanoma metabolic plasticity include AKT, BRAF, p14ARF, MYC, NRAS, phosphatidylinositol-4,5-bisphosphate 3 kinase catalytic subunit α (PIK3CA) and phosphatase and tensin homolog (PTEN) (14–20). Anyway, the remarkable metabolic flexibility and reprogramming of melanoma cells account for the impressive aggressiveness of CM and can also sustain resistance response to BRAF/MEK inhibitors (BRAFi/MEKi) and immunotherapy (2, 6). In this scenario the metabolic crosstalk between cancer cells and TME dramatically affects the metabolic choice, the growth and therapeutic resistance of CM (2). Therefore, in this article we discussed the metabolic plasticity of CM and the metabolic interactions of melanoma cells with TME, leading to tumor development, therapeutic resistance and immune escape. Finally, we highlighted the therapeutic strategies targeting the shift from glycolysis toward oxidative phosphorylation (OXPHOS).

## Glycolysis and Lactic Fermentation

Rapidly proliferating melanoma cells produce ATP and carbon precursors for cell growth and proliferation mainly through glycolysis and lactic fermentation, independently by oxygen levels. This process is named “Warburg effect” or aerobic glycolysis (21).

**Figure 1:**
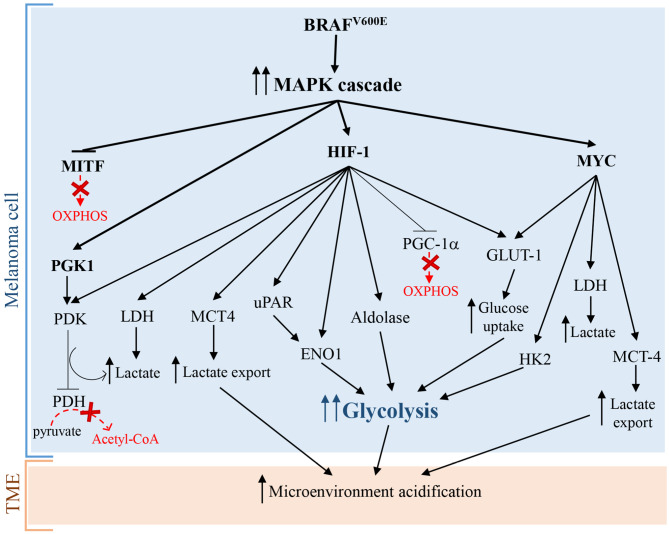
Warburg effect in melanoma cells caused by BRAF^V600E^ mutation leading to constitutive activation of MAPK pathway. Arrows indicate activation and T-bars show inhibition. Dotted red arrows, also indicated by a “red X,” symbolize inactive pathways, while solid T-bars and arrows indicate active signaling during Warburg effect.

Hypoxia-inducible factor 1 (HIF-1), that is a master regulator of numerous hypoxia-inducible genes and of glycolysis in melanoma, is usually inhibited during normoxia due to the rapid degradation of its subunit HIF-1α. However, many experimental evidence showed that HIF-1α and several of its target genes are strongly upregulated in melanoma cells not only during hypoxia, but also in normoxic conditions (22, 23). Additionally, under normoxia, melanoma cells can regulate and stabilize HIF-1α at the translational level, through mTOR and melanoma antigen-11 (MAGE11) (22), that is involved in the inhibition of prolyl hydroxylase domain protein 2 (PHD2), a HIF-1α negative regulator (24). Consequently, the upregulation and the protein stabilization of HIF-1α lead to the glycolysis induction in melanoma cells and to melanoma development and progression both in the presence and in the absence of oxygen (22, 25). Furthermore, it has been reported that the aberrant and constitutively activation of oncogenic signaling pathways, such as MAPK/ERK, phosphatidylinositol 3-kinase (PI3K)/AKT, mutated microphthalmia-associated transcription factor (MITF), endothelin dependent signaling, and ROS/NFkB pathways, are involved in the enhanced expression of HIF-1α in melanoma cells, independently by oxygen levels (22, 26–29). Interestingly, since MITF pathway activation and BRAF mutations, leading to MAPK signaling activation, occur, respectively, in about 10–20% (30) and 50% (31) of melanomas, it is possible to assume that the constitutive expression of HIF-1α is not a rare event in melanoma (32) and strictly correlates with melanoma aggressiveness and malignancy (23). In more detail, it has been reported that BRAF^V600E^ mutation, leading to the constitutive activation of ERK1/2 and MAPK pathway, sustains aerobic glycolysis through the activation of transcriptional factors, including HIF-1α and c-myc (22) (Figure 1). HIF-1α by interacting with HIF-1β, that generally is constitutively expressed (34), promotes the transcriptional activation of lactate dehydrogenase (LDH), aldolase, and enolase 1 (ENO1) (35) and consequently leads to an increase of glycolytic fluxes. In addition, HIF-1α turns on pyruvate dehydrogenase kinase (PDK), which prevents the entry of pyruvate in tricarboxylic acid (TCA) cycle by inhibiting pyruvate dehydrogenase (PDH) (33). More specifically, PDH converts pyruvate into acetyl-CoA in the mitochondria and the PDK-mediated inhibition of PDH leads to a lower consumption of pyruvate in the mitochondrion, and consequently, making a higher amount of pyruvate available in the cytosol. The increase of cytosolic pyruvate promotes a sustained lactic fermentation, consequently increasing lactate production (33). Persistent and sustained ERK1/2 activation, induced by mutant BRAF or KRAS, leads to mitochondrial translocation of phosphoglycerate kinase 1 (PGK1) and phosphorylation of PDK1, which in turn inactivates PDH, contributing to aerobic glycolytic switch in cancer (36). Further, glycolytic flux and glucose uptake are also stimulated by MYC, which transcriptionally activates LDH, glucose transporter 1 (GLUT-1), and hexokinase 2 (HK2) (37, 38).

Surprisingly, additional mechanisms promoting glycolysis in melanoma involve both the activation and the inhibition of the transcriptional factor and OXPHOS inducer MITF. As briefly stated before, the activation of MITF can promote the induction of HIF-1α, that is the master regulator of glycolysis (28). Interestingly, also MITF inhibition, via MAPK pathway, leads to glycolysis in melanoma cells. In particular, the constitutive activation of BRAF/MAPK pathway leads to the suppression of MITF, that thereby cannot activate the peroxisome proliferator-activated receptor γ 1-α (PGC1-α), an important driver of mitochondrial biogenesis and respiration. This leads to the metabolic switch toward glycolysis in melanoma cells (39). Even if these findings seem to be contradictory, they greatly highlight the complexity of melanoma pathogenesis: in fact the mutational status of melanoma cells and the microenvironmental signals can activate or inhibit specific molecular pathways in order to promote the “Warburg phenotype” in melanoma cells.

Furthermore, a sustained PI3K/AKT/mTOR signaling pathway positively acts on HIF-1α transcription and activity, triggering glycolysis through the synthesis of glycolytic enzymes (40, 41). Several mechanisms can activate PI3K/AKT/mTOR signaling in melanoma, including the loss of tumor suppressor PTEN functions, mutations in AKT and PIK3CA, and compensatory signaling through growth factor receptors (19, 42, 43). Interestingly, PTEN loss in melanoma cells increases extracellular acidification rate (ECAR), likely due to an increased production of lactate (44). Pyruvate kinase (PK) is the glycolytic enzyme converting phosphoenolpyruvate (PEP) to pyruvate in the last reaction of glycolysis. PKM2 is the most representative isoenzyme of the glycolytic enzyme PK. In cancer cells the isoform PKM2 is highly expressed (45) but is endowed with a low activity (46). More specifically, tyrosine kinases including FGFR1, BCR-ABL, and Jak2 phosphorylate glycolytic enzymes, such as PKM2. Tyrosine phosphorylation of glycolytic enzymes induces on one hand the activation of most glycolytic enzymes, thus increasing glycolytic rate, while on the other hand reduces the activity of PKM2, promoting paradoxically Warburg Effect (47). Rather than favoring the glycolytic fluxes with ATP production, the low PKM2 activity leads to an increased amount of glycolytic intermediates upstream PK reaction, providing precursors of several biological macromolecules, including nucleotides from glucose-6-phosphate (G6P), amino sugars, glycolipids and glycoproteins from fructose-6-phosphate (F6P), lipids from dihydrogenacetone-phosphate, serine from 3-phosphoglycerate, amino acids, and pyrimidines from PEP (45). Therefore, low PKM2 activity is a pivotal feature of cancer cells needing continuously precursors of biological macromolecules for their sustained and persistently elevated replicative rate (48). Interestingly, the phosphorylation of PKM2 by ERK rapidly transforms active tetrameric PKM2 into inactive monomers. The inactive monomeric form of PKM2 is able to translocate into the nucleus and induces the expression of many glycolytic enzyme genes, through epigenetic modifications of their promoters, by the phosphorylation of histone H3 (49).

The stimulation of aerobic glycolysis is highly depending on glucose uptake. Specifically, the transcription of the glucose transporter GLUT-1 is strictly regulated by HIF-1α (50, 51). The up-regulation of GLUT-1 is a common feature of the metabolic reprogramming in many tumors and can be associated with a high tumor grade (52–54). In particular, in CM compared to melanocytic nevi, it has been reported a higher GLUT-1 protein expression, which is positively associated with mitotic activity, melanoma progression, and metastasis (55, 56). Interestingly, the evaluation of GLUT-1 cellular localization, by immunohistochemistry, in 225 malignant melanomas and 175 benign nevi showed that GLUT-1 is frequently localized at cell membrane in melanoma, while in nevi this localization is infrequent (57). In other studies, GLUT-1 expression inversely correlated with overall survival (OS) or disease free survival (DFS) hence representing a tumor prognostic marker (58).

Lactic fermentation is a fundamental process, which converts pyruvate into lactate, reconstituting all the NAD+ that had been transformed into NADH during glycolysis. Consequently, to remove excess acid and to sustain glycolysis, lactate is secreted into the microenvironment, through monocarboxylate transporters (MCTs) (59). Interestingly, many cancers cells, including melanoma cells, take up lactate through MCT-1 and metabolize it, supplying the TCA cycle. Some evidences show that an increased transport of lactate correlates with worse outcomes (60). Therefore, lactate consumption could represent an useful biomarker of cancer progression. MCT-1 and MCT-4 represent the main bidirectional and ATP-independent transporters of lactate and related monocarboxylates through cell membrane, even though the directionality of transport is dependent on lactate and proton concentration gradients (61, 62). Pinheiro et al. demonstrated that in melanoma, the hyperexpression of GLUT-1 and MCT-4 correlated significantly with progression from primary to metastatic tumors. These data indicated that the glycolytic phenotype and lactate secretion synergistically act in promoting melanoma metastasis (63). In glycolytic tumor cells, HIF-1α and MYC upregulate MCT-4 to promote the secretion of lactate into the TME (64, 65). Recently, Tasdogan et al. showed that metabolic differences among melanoma cells confer a different ability to form metastases, depending on the function of the MCT-1 transporter (66). Specifically, by experiments of metabolites tracing, using^13^C-labeled nutrients, they identified efficient and inefficient melanoma metastasizers. Efficient melanoma metastasizers were characterized by high ability to uptake lactate, depending on MCT-1 expression. In addition, lactate uptake was strictly associated with a high enrichment in metabolites related to the TCA cycle (citrate, glutamate, and malate), suggesting that carbon atoms were transferred from lactate to TCA. Conversely, MCT-1 inhibition led to reduction of lactate uptake but was barely effective on primary tumors growth (66). Furthermore, inhibition of MCT-1 was linked to reduction of circulating melanoma cells and decreased CM metastatic capability in patient-derived xenografts and in mouse melanomas. Consistently with previous works, they found that inhibition of MCT-1 or MCT-4 in melanoma cells induces oxidative stress, through the inhibition of lactate export, and a reduced glycolysis (66). Additionally, clinical evidences further support the concept that melanoma mostly relies on glycolysis. For example, the positron emission tomography (PET) with an analog of glucose, i.e., the 2-deoxy-2-[fluorine-18]fluoro-D-glucose (^18^F-FDG), is an excellent imaging tool that exploits the glucose avidity of melanoma cells for the detection of widespread metastasis, for staging and restaging and for the evaluation of therapy response (67). Even if ^18^F-FDG PET has no role in early cutaneous melanoma (68), it can be considered a sensitive method superior to routine and conventional methods (i.e., ultrasound, radiography, histology or clinical examination, and follow-up, etc.) for the detection of distant metastases from malignant melanomas (69). Indeed, ^18^FDG-PET scanning shows 100% of sensitivity and 100% of accuracy for detecting visceral and abdominal nodal metastases, and superficial lymph node metastases, respectively (67). Furthermore, glycolytic melanoma cells produce high levels of LDH-5 that is the more effective isoenzyme in the catalysis of pyruvate to lactate, in order to produce ATP (70, 71). LDH-5, that reflects the Warburg phenotype in cancer cells, can be used as an accurate predictor of prognosis and response to treatments in melanoma patients. In fact, it has been reported that LDH-5 expression is easily detected both in histologic melanoma sections and in the serum of melanoma patients, and strongly correlates with prognosis. Particularly, translational studies reported that high LDH blood levels allow the identification of melanoma patients with worse prognosis (72) and that may not benefit from immunotherapy (2).

## Pentose Phosphate Pathway

The pentose phosphate pathway (PPP) sustains survival and growth of cancer cells through the generation of pentose phosphate sugars, which will be utilized for nucleic acid synthesis and will provide nicotinamide-adenine dinucleotide phosphate (NADPH). On its turn, NADPH will be essential for fatty acid (FA) synthesis and will sustain cell survival (73). The PPP is mainly regulated at glucose-6-phosphate dehydrogenase (G6PD) level, which acts as a “gatekeeper” of this pathway. G6PD catalyses the irreversible reaction of transformation of G6P into 6-phosphogluconolactone in a rate-determining step, generating NADPH (74). Subsequently, G6PD activity determines both the metabolic fate among glycolysis and PPP, and the oxidative PPP flux (75). The hyper-expression of G6PD is frequent in cancer cells and could be considered a biomarker of poor prognosis, indicating that G6PD has a fundamental role in tumorigenesis (76). Another important enzyme in the PPP, playing a pivotal role in melanoma proliferation and progression, is the transketolase (TKT). TKT converts excess of ribose-5-phosphate (R5P) into glyceradehyde-3-phosphate (G3P) and F6P through a number of reactions. Furthermore, G3P is metabolized also in glycolysis, and F6P can be converted into G6P that re-enters the oxidative PPP to produce further NADPH (77). Elevated TKT expression levels were reported in melanoma as well in lung, breast and prostate cancer cells. More specifically, exposure to UVA augments the proliferation of melanoma cells, by increasing the expression levels of TKT in melanoma (78).

## Oxidative Phosphorylation and KREBS Cycle

To generate ATP, melanoma cells adopt mainly glycolysis. In some cases, to cope energetic and metabolites demand, melanoma cells can also perform a massive OXPHOS. This metabolism is mainly driven by PGC1-α, which contributes to transcriptional induction of several mitochondrial genes (39, 79, 80) involved in specific mitochondrial processes including DNA replication, transcription, fission and fusion. In addition, hyper-expression of PGC1-α correlates with a decreased OS in patients with stage III melanoma (81) and with resistance to MAPK pathway inhibitors (MAPKi) (79, 80). Furthermore, PGC1-α-high expressing melanoma cells show a reduced sensitivity to reactive oxygen species (ROS), conferring an increased metastatic potential (81). Conversely, PGC1-α knockdown leads to the inhibition of ROS-scavenging gene expression, associated with an increased cell sensitivity to ROS (81) and the inhibition of metastatic spread of B16–F10 melanoma cells (82). MITF is a positive regulator of PGC1-α expression and other OXPHOS genes, including ATP5B, ATP5D (encoding for components of ATP synthase complex), CYC1 (encoding for cytochrome c1), NDUFA8, NDUFA9, NDUFB10, NDUFC2, NDUFS3 (encoding for components of NADH dehydrogenase complex), SDHB (encoding for succinate dehydrogenase subunit B) (39, 79, 83), while HIF-1α reduces PGC1-α expression levels by preventing the transcription of MITF (84, 85). AMP-activated protein kinase (AMPK) is an additional regulator of PGC1-α expression (86). In presence of a high cytoplasmic ratio of AMP/ATP, the kinase LKB1 phosphorylates and activates AMPK, which in its turn suppresses mTORC1 and inhibits anabolic reactions. Moreover, the activation of AMPK promotes mitochondrial gene expression *via* PGC1-α (86, 87). In glycolytic tumors, phosphorylation of ERK (pERK) prevents the activation of LKB1 and, consequently, reduces PGC1-α expression levels, inhibiting the typical response to energy deficiency (88).

The TCA cycle represents another mitochondrial pathway playing a pivotal role in tumor formation and progression. The TCA cycle occurs in the mitochondrial matrix and is an amphibolic pathway, in which multiple catabolic and anabolic pathways converge. In the last decade, it has been showed that several intermediates of Krebs cycle, including succinate, α-ketoglutarate, itaconate, fumarate, 2-hydroxyglutarate, are characterized by “non-metabolic” functions. These metabolites are involved in epigenetic modifications or post-translational protein modifications, that affect the immune response and contribute to pathological conditions, such as initiation and progression of carcinogenesis (89). α-ketoglutarate and succinate levels can regulate the activity of HIF-1α via prolyl hydroxylases (PHDs), promoting a metabolic switch from OXPHOS to glycolysis (90). Specifically, PHD uses molecular oxygen to hydroxylate HIF-1α, at specific residues of proline. Hydroxylation recruits on HIF-1α the protein Von Hippel-Lindau (VHL) E3 ubiquitin ligase, which ubiquitinates and subsequently promotes the proteasome-dependent degradation of HIF-1α (91). Interestingly, a recent work (92) shows that MITF, through the transcriptional regulation of SDHB, contributes to prolong hypoxia response. Specifically, under hypoxia, by the action of BHLHE40/DEC1, the levels of MITF expression and activity decrease (85). Consequently, because SDHB converts succinate in fumarate, the levels of succinate increase. On its turn, succinate inhibits PHD, preventing HIF-1α degradation (90). In addition, increased amount of succinate can affect the regulation of multiple enzymes through the process of succinylation (93). It has been shown that cytoplasmic aspartate levels can promote tumor progression in melanoma, through the suppression of arginosuccinate synthetase 1 (ASS1), which, in the urea cycle, converts aspartate into arginosuccinate. The increase of intracellular levels of aspartate activates the carbamoyl phosphate synthetase II (CAD), which, consequently, leads to an increased synthesis of nucleotides and promotes melanoma cell proliferation (94).

Glutamine represents the main metabolite able to replenish the TCA cycle of precursors, required for the synthesis of fats, nucleic acids and amino acids (95). Furthermore, glutamine metabolism provides energy and is pivotal for cellular redox homeostasis (96). Differently from melanoma, other glycolytic tumors replenish the TCA cycle of precursors through the action of enzyme pyruvate carboxylase which produces oxaloacetate from pyruvate (97). Interestingly, in melanoma the contribution of pyruvate carboxylase to the TCA cycle is very low (21, 98, 99). After entering the cell through the glutamine receptor SLC1A5, glutamine is deaminated to glutamate by the action of cytosolic glutaminase (6). Consequently, glutamate is converted into α-ketoglutarate, through reactions catalyzed by either glutamate dehydrogenase 1 (GDH1) or mitochondrial alanine and aspartate aminotransferase (GOT2 and GPT2) and enters the TCA cycle. Interestingly, through a reductive carboxylation of α-ketoglutarate, tumor cells are able to reverse Krebs cycle, thereby increasing the amount of citrate to be used for FA synthesis. Of note, under low presence of oxygen, α-ketoglutarate, which derives from deamination of glutamate, provides over one-third of total citrate necessary for FA synthesis (21). The main enzymes required for the production of citrate through the carboxylation of α-ketoglutarate are cytosolic and mitochondrial isocitrate dehydrogenases, respectively IDH1 and IDH2. Some works reported that mutations in these genes sporadically arise in melanoma (83, 84) and cause a growth advantage to melanoma cell lines bearing BRAF mutations (85).

## Fatty Acid Oxidation

In the last years, fatty acid oxidation (FAO) in cancer has been extensively studied and growing evidences show its contribution in melanoma progression. Comparative analyses between melanoma cells and benign nevi show that carnitine palmitoyltransferase 2 (CPT) 2, an enzyme critical for translocation of long-chain Fas, is one of the most upregulated gene in melanoma (100). Interestingly, melanoma cells treated with MAPKi showed an increase of CD36 levels and fatty acid oxidation (FAO) levels in a manner dependent by peroxisome proliferator-activated receptor (PPAR-α) and CPT1A (101). Of note, the sustained FAO is essential for survival of BRAF^V600E^-mutant melanoma cells, under the MAPKi-induced metabolic stress prior to acquiring drug resistance (101). Being the metastasis formation a process that require a huge amount of nutrients, FAs can provide an ATP boost for the dissemination of tumor cells. In addition, FAs can provide acetyl-CoA, which, in the TCA cycle, is essential for citrate formation and, consequently, for NADPH production *via* IDH1, participating to the redox balance in tumor cells (102). Interestingly, some proteins that bind and process lipids, including phospholipase D3 (PLD3), inositol triphosphate protein kinase B (ITPKB), inositol triphosphate receptor 3 (ITPR3), fatty acid binding protein 3 (FABP3), have been found strongly upregulated in melanoma (100). In addition, a recent comparative analysis of proteome of melanoma cells revealed a higher OXPHOS and lipid metabolism in melanoma cells “responder” to immunotherapy (103). More in detail, Harel et al. showed that a higher OXPHOS and lipid metabolism augment the antigen presentation of melanoma cells, through the increase of MHC proteins expression (HLA-A, HLA-C, and B2M which consist MHCI, and CD74, a chaperone of MHCII), of several factors involved in the antigen processing and presentation machinery (including TAP1 and TAP2 which are peptide antigen transporters, TAPBP which acts as a bridge between the MHC and the peptide transporters), and PSME1, a component of proteasome. Consequently, the higher expression of MHC molecules promotes a better response upon immunotherapy by T cells (103).

## Melanoma Microenvironment Acidification and Its Influence on Melanoma Growth and Therapeutic Resistance

**Figure 2:**
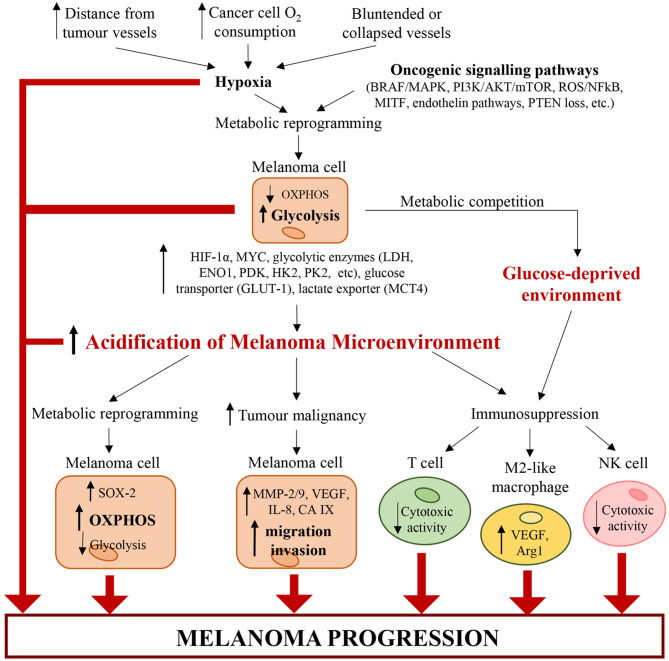
Alterations of cancer and immune cells associated with melanoma microenvironment acidification. Hypoxia (caused by increasing distance from tumor vessels, structural abnormalities of tumor vasculature and high cancer cell O_2_ consumption) and genetic mutations (such as BRAF^V600E^, loss of PTEN, etc.) trigger a metabolic reprogramming in melanoma cells, which increase glycolysis. Glycolytic melanoma cells promote an abnormal extracellular accumulation of lactate and protons and thus induce the acidification of TME. In turn, the acidic TME triggers a metabolic reprogramming in melanoma cells, which increase OXPHOS and decrease glycolysis. Additionally, tumor acidity increases melanoma malignancy by enhancing the migratory and invasive capability of melanoma cells and their capability to produce proteases (such as MMP-2/9, cathepsin, etc.) and pro-angiogenic factors (such as VEGF-A, IL-8, etc.). Acidity generates an immunosuppressive milieu, where T cells and NK cells lose their cytotoxic activity, and macrophages acquire the protumorigenic M2-like phenotype. Also glycolytic melanoma cells contribute to the generation of this immunosuppressive environment. In fact, by mediating glucose restriction, melanoma cells alter T cell metabolism and reduce their cytotoxic activity. Therefore, the pH and oxygen gradients in the tumor mass lead to molecular and metabolic changes in cancer and immune cells, which work together and cooperate to sustain melanoma progression.

The acidification of microenvironment is a hallmark of CM (2) (Figure 2). In fact, pH gradient of melanoma cells is totally different respect to that of normal cells (104). Cancer cells display an intracellular pH (pHi) > 7.4 and extracellular pH (pHe) ranging from 6.7 to 7.1. Conversely, normal cells display pHi of about 7.2 and pHe of about 7.4 (104). The extracellular acidosis is linked to metabolic changes of melanoma cells and angiogenesis (2). The alterations in cancer cell metabolism are represented mainly by upregulation of glycolysis leading to protons and LDHA-dependent lactate generation (10). Both protons and lactate are transported out of cancer cells by MCT-4, proton exchangers and transporters, in order to elude intracellular acidosis (10, 105). It is noteworthy that stromal cells can take extracellular lactate to produce pyruvate, successively secreted to furnish melanoma cells (106). Furthermore, also PPP and glutaminolysis sustain microenvironment acidification through secretion of carbon dioxide (CO_2_) (2).

Angiogenesis process generates new blood vessels from already formed vessels (107). In particular, the vasculature affects dramatically the metabolism of solid tumors because the distance of cancer cells from new vessels influences metabolic option between glycolysis and OXPHOS (108). Furthermore, angiogenesis and vascular network, characterized by both hypervascularisation and hypovascularisation, influence the tumorigenic, mitogenic and metastasizing VGP step of CM (2, 3, 109, 110). Both hypervascularisation and hypovascularisation depend on the loss of homeostasis between pro-angiogenic and anti-angiogenic factors (111). This pathological condition leads to migration and proliferation of endothelial cells, whose excess sustains the development of disorganized and hyperpermeable blood vessels (111). Additionally, blood vessels of TME are also compressed by cancer cells. These structural alterations of tumor blood vessels lead to an increase of resistance to blood flow associated with a reduction of blood supply (111). Furthermore, in highly aggressive melanomas, cancer cells can acquire an endothelial phenotype which enables their participation in angiogenesis (112). It is noteworthy that hyperpermeability of cancer vessels generates the lack of pressure gradient modulating the circulation of both fluid and macromolecules and sustains hypoxia by hindering the transport of oxygen. The hypoxia, generated also by high oxygen consumption of cancer and endothelial cells (111), sustains acidosis through up-regulation of glycolytic pathway mostly linked to the stabilization of HIF-1α, the principal inductor of aerobic glycolysis in cancer (113, 114). Therefore, HIF triggers up-regulation of plasma membrane transporters, exchangers, pumps and enzymes, all of which keep pHi of cancer cells around neutral values or even lightly alkaline (114, 115). However, the acidification of melanoma microenvironment can be transient or chronic and is associated with alterations of cancer cells and, as discussed below, of immune cells (Figure 2). Furthermore, a recent work of Acker's group indicates that metabolic alterations of melanoma cells, hypoxia, HIF-1 and microenvironment acidosis are regulated by a common positive feedback (116). HIF-1α not only regulates both anaerobic and aerobic glycolysis in melanoma cells, but also enhances the expression of genes involved in both tumor invasion and glycolysis. In particular, under hypoxia (117, 118) and normoxia (119), HIF-1α can increase the expression of urokinase plasminogen activator receptor (uPAR), which in turn leads to a glycolytic and invasive phenotype in melanoma cells in a EGFR-dependent manner with involvement of the PI3K/mTOR/HIF-1α pathway (117–119). The binding of the urokinase plasminogen activator (uPA) to its receptor uPAR, expressed in one-third of melanomas (120), sustains the expression of the extracellular matrix metalloproteinases inducer (EMPPRIN) and of ENO1, which both connect lactate homeostasis and glycolysis with the invasive phenotype of melanoma cells (119). Therefore, uPA/uPAR system in melanoma cells could be a molecular connection between invasion capability and glycolytic metabolism (119).

Furthermore, Laurenzana et al. showed that there is a strong connection between uPAR levels in BRAF mutant melanoma cells and response to BRAF inhibition (121). In fact, melanoma cells, expressing different levels of uPAR, show variable responsiveness to Vemurafenib. These experimental evidences suggest that uPAR levels could predict outcome of targeted therapy in patients affected by BRAF mutant melanoma (121). CM microenvironment acidification can confer a growth advantage to cancer cells by selecting cells resistant to acidic conditions (122), even if cancer cells can respond to acidic microenvironment in different ways. In particular, Peppicelli et al. demonstrated that acidic microenvironment may trigger in melanoma cells a metabolic shift toward OXPHOS and mesenchymal phenotype, associated with high invasiveness and pro-metastatic property (13).

Biguanide metformin, that is a molecule used in the treatment of type 2 diabetes, specifically inhibits the mitochondrial respiratory chain (MRC) complex 1. It leads to NADH oxidation decrease and to reduction of both proton gradient across the inner mitochondrial membrane and oxygen consumption rate (123). Metformin treatment inhibits both epithelial-mesenchymal transition (EMT) markers and OXPHOS at concentration of 10 mM, which is non-toxic for cancer cells grown in a standard pH medium. Furthermore, this treatment inhibits remarkably proliferation and colony formation of acidic melanoma cells, grown in acidic microenvironment. Therefore, the capacity of metformin to hinder EMT and OXPHOS supports the supplement of metformin to therapy of advanced melanoma (13, 124).

Transient treatment of A375-M6 melanoma cells with acidic medium increases expression of SOX2 with respect to control cancer cells grown in standard medium (125). Extracellular acidosis induces a metabolic switch toward OXPHOS and a concurrent slowdown of acidic cancer cells to a more glycolytic metabolism (126). The silencing of SOX2 gene shifts the metabolism of acidic melanoma cells toward glycolysis, thus making cancer cells less vulnerable to metformin treatment (125, 126). SOX2 is a transcription factor regulating, under acidic condition, the metabolic shift toward OXPHOS and downregulates HIF-1α by binding to its promoter (125, 126). Recent studies highlighted that the extracellular acidity of melanoma TME may provide an environment sustaining dormancy of melanoma cells, which exhibit low replication rate, high resistance to apoptosis and autophagy (127). Tumor dormancy is an important process implicated in tumor immune escape and drug resistance (128, 129). Jia et al. showed that low levels of SOX2 expression are linked to cycle arrest, melanoma cell stemness and tumor dormancy leading long-term tumor survival, and relapse (130).

Other studies showed that acidosis induces apoptosis and autophagic pathway (131–133). Böhme et al. showed that chronic acidosis triggers in melanoma cells a senescence-like phenotype with MITF^low^/AXL^high^ signature and cellular translation reprogramming. This phenotype, induced by extracellular acidosis, is associated with therapeutic outcome in CM (134).

Transient or chronic extracellular acidification increases carbonic anhydrase IX (CAIX) expression (135). CAIX is a transmembrane enzyme that is an important regulator of cancer cells pHi (136, 137). CAIX enzymatic activity affects viability of acidic cancer cells in CM, and its inhibition could represent a new therapeutic strategy (135). In particular, Chafe et al. analyzed the expression of CAIX in a cohort of 449 patients affected by CM. They showed that CAIX levels are linked to worse OS (138). CAIX inhibition, through SLC-0011 treatment, reduces extracellular acidosis and improves the response to anti–programmed cell death protein 1 (PD-1) and anti–cytotoxic T-lymphocyte-associated protein-4 (CTLA-4) blockade. These effects are associated with reduction of melanoma growth (138).

Lipid rafts are plasma membrane subdomains containing high concentrations of cholesterol and glycosphingolipids (139). V-type H^+^-ATPases enzymes are proton pumps, present in lipid rafts, and whose plasma membrane overexpression is correlated with cancer metastasis (140–142). The enzymatic activity of these H^+^ pumps supports extracellular acidosis that sustains the activity of proteolytic enzymes, such as metalloproteinases (MMPs) and cathepsins, and promotes drug resistance and metastasis (104). Inhibition of V-ATPases in melanoma cells, by using the plant-derived monoterpene Myrtenal, hampers the electrochemical H+ gradient across the cell membranes, triggers cell death and decreases tumor cell migration and invasion *in vitro* (143). Moreover, V-ATPases inhibition reduces metastasis *in vivo* (143). Acidic microenvironment induces cancer cells to synthesize and secrete proteases such as MMP9 and 2, cathepsin B and L, all of which can degrade extracellular matrix (ECM) proteins (122). Furthermore, melanoma cells increase the secretion of the proangiogenic factors VEGF-A and IL-8 (144). All these experimental evidences strongly support a relation between extracellular acidosis and malignant progression, higher invasion, and metastasis of melanoma cells. In particular, the dissemination of CM strongly depends on the spreading and propagation of cancer cells to lymphatic vessels and regional lymph nodes, respectively (145). The lymph nodes are the prevalent site of CM metastasis. Both in A375P melanoma cell line and in melanoma cells derived from a human metastatic lesion, extracellular acidosis induces the expression of VEGF-C. This growth factor, that is secreted by both melanoma cells and tumor associated macrophages (TAMs), induces dramatically lymphoangiogenesis (146).

Exosomes could have a significant role in solid tumor progression because they have an unlimited access to the lymphatic system and blood vessels (147–149). Boussadia et al. showed that acidic microenvironment sustains exosome secretion of melanoma cells (150). Consequently, extracellular acidosis trough a massive release and intra-tumoral uptake of exosomes, triggers a more malignant and metastatic phenotype in melanoma cells. In fact, pH naïve melanoma cells, exposed to exosomes generated in an acidic medium, develop migratory and invasive capability probably associated with transfer of metastatic exosomal proteins, sustaining cell motility and angiogenesis (150). Furthermore, exosomes from melanoma cells could contribute to extracellular acidification, by interacting with normal stromal fibroblasts located in distant sites from primary tumors (151). In particular, exposure of human adult dermal fibroblasts to human melanoma-derived exosomes leads to fibroblast metabolic reprogramming associated with increase of aerobic glycolysis, decrease of OXPHOS and extracellular acidification induction (151). In particular, the activity of exosomal miR-155 and miR-210 regulates upregulation of aerobic glycolysis in fibroblasts. Therefore, cancer cell exosomes could influence stromal cell metabolism, thus contributing to the generation of a pre-metastatic niche that sustains metastatic process (151).

It is known that melanoma microenvironment acidification hinders immunotherapy response (2). Additionally, LDH serum level is a well-known prognostic factor of survival in CM (2, 152). In fact, LDH levels significantly affect response, progression-free survival (PFS) and OS of CM patients treated with antibodies targeting CTLA-4 (ipilimumab) or PD-1 (nivolumab, pembrolizumab), or with ipilimumab plus nivolumab combined therapy (152). Lactate can contribute to tumor escape from immune response by impairing cytotoxic T lymphocytes (CTLs) metabolism and function (153). High levels of lactate are linked to a significant decrease of CD8+ T and NK cell number and activity, both in *vitro* and in *vivo* (154). Furthermore, Collegio et al. showed in murine experimental model that lactate induces in macrophages a pro-tumoral M2-like phenotype, characterized by the induction of VEGF and arginase 1 (Arg1) expression (155). Therefore, the efficacy of immunotherapy could be improved by counteracting microenvironment acidification and lactate extracellular accumulation. As stated before, CAIX inhibition though SLC-0011 treatment decreases the acidification of melanoma microenvironment. SLC-0011 treatment combined with immune-checkpoint inhibitors enhances anti–PD-1 and anti–CTLA-4 blockade effectiveness (138). Another possibility to ameliorate immunotherapy outcome could be represented by LDH inhibitors, which however cannot be used in melanoma therapy, because preclinical analyses of anticancer activity have demonstrated their low therapeutic effectiveness associated with harmful side effects (156).

Diclofenac and lumiracoxib are non-steroidal anti-inflammatory drugs (NSAIDs), approved for clinical use, that display structural similitude (157). In particular, diclofenac induces apoptosis, associated with mitochondrial dysfunction, and restrains both glucose metabolism and MYC expression in melanoma (158, 159). It is noteworthy that both NSAIDs could be utilized to counteract lactate extracellular accumulation (157).

## Metabolic Crosstalk Between Melanoma Cells and TME

The TME of solid tumors such as CM is extremely complex. In fact, it includes ECM molecules, represented by laminin and collagen, growth factors, including VEGF, nutrients, such as glucose, blood and lymphatic tumor vessels, various concentrations of oxygen, cancer and stromal cells, that influence each other to sustain tumor growth, progression and metastasis (9, 160, 161). Non-cancer stromal cells are represented by endothelial cells, pericytes, immune cells, fibroblasts, fibroblast aggregates, myofibroblasts, cancer associated fibroblasts (CAFs), activated adipocytes, and mesenchymal stem cells (MSCs) (9, 162–165). In the complex, melanoma microenvironment represents a niche produced and regulated by the bidirectional interactions of melanoma cells with surrounding cells, tumor vessels and ECM (166). This crosstalk dramatically influences the development of disease and therapeutic resistance (2). In solid tumor cancer cells trigger a constitutive wound healing response that provokes an unregulated inflammation and a constitutive stroma activation (167). In particular, during melanoma growth and development, cancer tissue recruits and activates host tissue cells which sustain the development and progression of tumor, by supporting the metabolism of cancer cells (167). Therefore, the several activated stromal cells can differentiate into macrophages, mast cells, adipocytes and CAFs, which in turn begin to secrete cytokines, induce a turnover of ECM thus playing an important role in tumor growth (167).

### Metabolic Crosstalk Between Fibroblasts and Melanoma Cells

Fibroblasts, fibroblast aggregates, myofibroblasts and CAFs represent important constituents of melanoma stromal microenvironment (163, 165, 168). It is known that normal dermal fibroblasts, before CAF differentiation, hamper melanoma formation at its early stage (169). Conversely, senescent fibroblasts support melanoma growth (170). During melanoma development fibroblast aggregates are formed in dermis and their interaction with melanoma cells leads to fibroblast reprogramming linked to CAF differentiation (163). Anyway during melanoma development and progression, skin fibroblasts, through direct contact with malignant melanocytes, and/or the stimulation by humoral mediators and ROS, undergo a metabolic transformation which leads to fibroblast constitutive activation and thus CAF differentiation (9).

Direct contact between melanoma cells and fibroblasts are modulated by cadherins and connexins (171). In CM cancer cells proliferate, penetrate basement membrane and infiltrate into corium. Furthermore, a change from E-cadherin to N-cadherin expression, during melanoma development, not only frees cancer cells from epidermal keratinocytes, but also provides new adhesive characteristics (171). Via N-cadherin and gap junctions regulated by connexins, melanoma cells interact with N-cadherin-positive fibroblasts (171). In particular, connexins-mediated gap junctions regulate TME interactions between melanoma cells among each other and with the stromal cells (172). Connexins belong to a family of transmembrane proteins building gap junctions. These proteins are essential for gap junctional intercellular communication (GJIC) by exchange small molecules such as ions, signaling molecules (cAMP, ATP) and amino acids (172). Furthermore, GJIC mediates also miRNA transfer (173). The expression of connexin-43 (Cx43), over the last few years, has been associated with cancer recurrence, metastatic spread and poor survival (174). Furthermore, it is conceivable that Cx43 mediated connection could couple metabolic profile of melanoma tissue with regulation of cancer growth. In fact, Cx43 hemi channel regulates proliferation by regulating intracellular ATP and Ca^2+^ levels (175). Notably, inhibition of Cx43 channel activity sustains melanoma cell proliferation, while overexpression of Cx43 increases gap junction GJ coupling and reduces cell growth. Furthermore, Cx43 overexpression in FMS human melanoma cell lines increases apoptosis and is linked to a decrease of melanoma growth and metastatic capability (176).

Paracrine interaction, leading to CAF differentiation, is affected by tumor cell-derived growth factors and cytokines such as transforming growth factor β (TGF-β), epidermal growth factor (EGF), platelet-derived growth factor α/β (PDGFα/β), basic fibroblast growth factor (bFGF, also known as FGF2), interleukin 6 (IL-6), and interleukin 1β (IL-1β) (7). In particular, TGF-β influences significantly CAF differentiation, because it sustains the increase of fibroblast ROS that modulate α-SMA expression which is a marker of CAFs (7). Expression of Nodal, one of the member of the TGF superfamily, is positively correlated with melanoma (177). Nodal induces, together with Snail and TGF-β signaling pathway activation, the differentiation of normal fibroblasts into CAFs which sustain melanoma growth both *in vitro* and *in vivo* (177).

It is known that ROS are important modulators of the interplay between fibroblasts and cancer cells. ROS homeostasis is affected by the balance between ROS generation and both enzymatic and non-enzymatic antioxidant systems: the destroying of this equilibrium drives oxidative stress, which participates in tumor development (9). In particular, ROS production can be the result of melanocytes metabolism, melanin metabolism and UV radiation, altered metabolism of transformed cells (178). Melanoma cells derive from epidermal melanocytes in skin, which is a moderately hypoxic tissue (179). Melanocytes produce ROS through mitochondria, melanosomes, NADPH oxidase (NOX) family enzymes, different arachidonic acid oxygenase activities, and nitric oxide synthase (NOS) activity/uncoupling (179). Furthermore, melanocytes are highly exposed to oxidative stress because of the pro-oxidant state linked to melanin production, and the intrinsic antioxidant defenses weakened by pathologic conditions (179). In particular, it is noteworthy that melanin protects melanocytes by adsorbing UV radiation, but its synthesis is linked to higher levels of intracellular ROS that may sustain carcinogenesis (180). Furthermore, even if melanocytes are protected by endogenous melanin which absorbs ROS generated by UV radiation, melanin can be oxidized by exposure at higher UV doses, thus leading to ROS generation (180). However, both acute and chronic UV radiations trigger the skin to produce ROS, whose impact is exacerbated by the relative deficiency of melanocytes in the repair of oxidative DNA lesions (180). It is remarkable that hydrogen peroxide (H_2_O_2_) is a ROS regulating tyrosinase, the key enzyme for melanin synthesis in normal melanocytes and melanoma cells (181). Tumor hypoxia can contribute to ROS production by triggering the mitochondrial production of superoxide anion that is transformed to H_2_O_2_ by SOD2 activity. In particular, H_2_O_2_ is a very stable and permeable ROS, that can pass through both mitochondrial and cell membranes. It is the principal ROS in the regulation of signaling transduction pathways and modulates significantly the behavior of non-cancer and cancer cells (9, 182, 183). In solid tumors microenvironment, cancer cells generate high levels of ROS associated with mitochondrial dysfunction, upregulation of NOX-1 and NOX-4, and modifications of antioxidant enzymes (7). The mitochondrial dysfunction leads to the shift toward aerobic glycolysis, named as stated before “Warburg effect,” which is an early step of carcinogenesis, and can also arise before the development of hypoxia. In cancer cells both “Warburg effect” and mitochondrial malfunctioning augment lactate and ROS levels and reduce antioxidant molecules (9). There are many works that showed the high level of oxidative stress in melanoma cells *in vitro* (184–188). However, even if it is possible to suppose that CM is a reactive oxygen driver tumor, the association between oxidative stress and human CM development and progression is still less studied (179). Anyway, the contribution of NOX-4 to transformed phenotype of melanoma cells by modulating G2-M cell cycle progression, suggests that specific signals of NOX family enzymes affect CM development (189). Furthermore, Ribeiro-Pereira et al. showed that ROS produced by the NOX, most likely NOX4, can sustain melanoma cells through the focal adhesion kinase (FAK) pathway, and thus maintain adhesion contacts and cell viability (190). ROS can trigger a cascade of intra- and intercellular signals driving metabolic reprogramming of both cancer cells and fibroblasts, and CAF differentiation. In particular, H_2_O_2_ from cancer cells triggers in CAFs an oxidative stress, connected with decrease of mitochondrial function and increase of both glucose uptake and ROS levels. Therefore, ROS produce a reactive microenvironment, where the energy needed for cancer cell proliferation is sustained by constitutively activated CAFs (9). Compared with normal fibroblasts, CAFs are characterized by slower proliferation rate, increased migratory capability, and resistance to apoptotic stimuli (2, 7, 163).

It is noteworthy that solid tumors like melanoma represent a heterogeneous metabolic environment, where the interaction between cancer cells and cancer microenvironment leads to a form of “parasitic” cancer metabolism (191). This metabolic model has been called by Lisanti's group “two-compartment tumor metabolism” or the “autophagic tumor stroma model of cancer” (192). The “autophagic tumor stroma model of cancer” is associated with clinical outcome in many tumors such as melanoma. In fact, in lymph node metastases of malignant melanoma loss of stromal caveolin 1 (Cav-1) expression, that is a marker of autophagy, glycolysis, and oxidative stress, strongly predicts clinical outcome (7, 193). Furthermore, the involvement of CAFs in melanoma dissemination is confirmed by study analyzing the clinical importance of Cav-1 expression in CM. In particular, loss of Cav-1 in CAFs could sustain metastatic process by producing a glycolytic microenvironment (193). Catabolic CAFs satisfy high-energy demands of cancer cells and sustain anabolic cancer growth, through secretion of considerable quantity of energy-rich fuels, like L-lactate, ketone bodies, glutamine, and free FAs (7, 191). Particularly, CAFs from human melanomas and colon cancers, characterized by high levels of glycolytic activity, glucose uptake, lactate production, glycolytic enzyme expression and decrease of oxygen consumption, support the CAF catabolic model (194). IDH3α, a key enzyme in the TCA cycle, regulates metabolic switch toward aerobic glycolysis in CAFs from melanomas and colon cancers. IDH3α downregulation in CAFs increases HIF-1α levels (194).

The horizontal transfer of mitochondria is another important mechanism that connects stromal and tumor cells (195, 196). *In vivo* studies performed by using melanoma cells without mitochondrial DNA (B16ρ^0^ cells) showed that mouse stromal cells can transfer whole and intact mitochondria in B16ρ^0^ cells, which consequently re-acquire respiratory function and the capability to form tumors efficiently. It is important to note that tumor cells without mitochondrial DNA form tumors with a very long delay with respect to their parental cells. Therefore, altogether these data suggest that even if many tumor cells rely mostly on the glycolytic metabolism, they also need mitochondrial respiration for promoting tumor formation and progression successfully and efficiently (196).

### Adipocytes in the Metabolic Crosstalk With Melanoma Cells

Metastatic melanomas often grow in subcutaneous tissues and can be associated with a poor prognosis (197). The subcutaneous tissues contain mainly adipocytes which can promote tumor progression (197–199). Zhang et al. showed a mechanism associated with the capability of adipocytes to sustain melanoma progression and demonstrated a new therapeutic target of melanoma microenvironment (197). In particular, they showed that adipocytes induce *in vitro* proliferation and invasion of adjacent melanoma cells by transferring lipids to cancer cells and thus modifying the metabolism of melanoma cells (197). The lipids, used by melanoma cells in the β-oxidation pathway, decrease the dependence on *de novo* lipogenesis. Furthermore, the transfer of lipids from adipocytes to cancer cells is regulated by FATP/SLC27A family of lipid transporters, which localize on cancer cell surface. Melanoma cells overexpress FATP1/SLC27A1 that, in transgenic zebrafish experimental model, work together with BRAF^V600E^ in sustaining CM development. Inhibition of Fatty Acid Transporter Proteins (FATPs), through the small-molecule inhibitor Lipofermata, decreases melanoma lipid uptake, invasion, and growth (197). Adipocytes secrete large amounts of exosomes, which are then taken up by melanoma cells, thus supporting migratory and invasive capability (200). These exosomes contain protein regulating and inducing FAO in melanoma cells. Furthermore, in obese humans, both the number of adipocytes-secreted exosomes and their influence on FAO-dependent cell migration are dramatically increased. These experimental evidences might in part offer an explanation for poorer prognosis of obese melanoma patients (200).

### Metabolic Interplay Between Immune and Melanoma Cells

It is known that in TME the crosstalk between melanoma and immune cells affects immune response (201). In particular, high metabolic plasticity of melanoma cells and changes in melanoma microenvironment can lead to the activation of immune escape mechanisms and formation of an immunosuppressive niche, hindering immunotherapy effectiveness (2, 202–204). The upregulation of glycolysis associated with remarkable secretion of lactate and extracellular acidosis results in dramatic changes of stromal immune cells. In this scenario T cells (205) and their metabolic crosstalk with cancer cells influence significantly the immune homeostasis of melanoma microenvironment and response to immunotherapy (2). The upregulation of glycolysis in melanoma cells produces a microenvironment lacking glucose, where tumor cells and tumor-infiltrating lymphocytes (TILs) are competitor for glucose uptake. In fact, intra-tumoral CD4 T cells display indicators of glucose deprivation and decreased anti-tumor effector functions, indicating that a glucose-poor TME might participate in TIL exhaustion (206). In particular, TILs exhibit an increased expression of several “anergy” signature genes, and a decreased rate of glycolysis linked to a reduction of the glycolytic metabolite PEP. In particular, PEP reduction leads to defects in both Ca^2+^- nuclear factor of activated T cells (NFAT) signaling and T cell activation by increasing sarco/ER Ca2+-ATPase (SERCA)-mediated Ca2+ re-uptake, thereby suppressing CD4+ T cell-mediated immune surveillance (206). Notably, metabolic reprogramming of TILs, by overexpression of phosphoenolpyruvate carboxykinase 1 (PCK1), that converts oxaloacetate (OAA) into PEP, increases their anti-tumor activities in the glucose-deprived TME (206). It is noteworthy that in murine melanoma experimental model, CD8+ TILs, which interact with a hypoglycemic and hypoxic TME, maintain their anti-tumor immune surveillance by shifting their metabolism toward OXPHOS, through the PPAR-α signaling activation and increasing FA catabolism (207). OXPHOS induction in TILs improves efficacy of PD-1 blockade therapy in murine experimental models (208). Additionally, the increase of FA catabolism in TILs might improve cancer immunotherapy in patients affected by tumors with low glucose content (207). Glycolysis satisfies the high energetic demand of activated T cells for their proliferation and cytokine synthesis, and the efficient secretion of lactate through MCT-1 allows to continue glycolysis in T cells. However, during melanoma progression, the high levels of lactate in TME hinder lactate export through MCT-1 from human CTLs. The lactate accumulated in CTLs inhibits their proliferation, cytokine production and decreases their cytotoxic activity. In particular, lactate decreases IL-2 and interferon γ (IFNγ) levels in CTLs (153). Furthermore, it is conceivable that the consequence of lactate accumulation on the immune system is dependent on the acidification rather than on the lactate itself (153, 209–211).

Arginine metabolism contributes to tumor progression and immunoescape. Arginine can be synthesized in two steps through the action of two tightly coupled enzymes, argininosuccinate synthetase (ASS) and argininosuccinate lyase (ASL). In particular, ASS catalyses the conversion of citrulline and aspartic acid to argininosuccinate, which is subsequently transformed into arginine and fumaric acid by ASL (212). Several tumors such as malignant melanoma, are arginine auxotrophic. Downregulation of the enzyme ASS, a well-known rate-limiting step in arginine synthesis, induces an intrinsic dependence on extracellular arginine caused by incapacity to synthesize arginine for growth (213). Therefore, high microenvironmental arginine uptake by cancer cells leaves little for T cells and drives reduction of their proliferation and survival (202). Furthermore, arginine is a precursor of nitric oxide (NO) whose level is a prognostic marker of cancer outcome for melanoma patients (214). NO can promote melanoma development through its immunosuppression activity, inhibition of apoptosis, stimulation of protumorigenic cytokines, activation of TAMs and alteration of angiogenic processes (214). Furthermore, NO can react with superoxide anions to yield peroxynitrite, which induces apoptosis in T cells (202). It is remarkable that arginine metabolism significantly contributes to inflammatory tumor environment (215). In fact, NO activates cyclooxygenase-2 (COX-2) and other inflammatory factors and thus generates a pro-oxidant microenvironment sustaining cancer cell growth and suppressing anti-tumor immunity (215). Additionally, NO has an anti-apoptotic influence in human melanoma cells, and the expression of inducible nitric oxide synthase (iNOS), that produces NO, is linked to worse survival in patients with Stage III melanoma (216).

## Hallmarks of Therapies Targeting the Shift of Melanoma from Glycolysis Toward OXPHOS

BRAF mutations induce melanoma cell proliferation, mitochondrial alterations (39) and fragmentation (217), and consequently the metabolic switch from OXPHOS toward glycolysis (218). Despite their glycolytic phenotype, melanoma cells harboring BRAF^V600E^ mutation exhibit high metabolic flexibility, which thus can represent a promising and successful target in melanoma therapy. After an initial cancer regression in BRAFi/MEKi-treated patients, a subset of melanoma cells can acquire a metabolic drug-resistant phenotype, characterized by the enhancement of mitochondrial biogenesis, mitochondrial activity and mitochondrial content (2, 219, 220). The metabolic reprogramming from glycolysis toward OXPHOS is an adaptive response, which allows melanoma cells to provide ATP levels and avoid cell death process despite the inhibition of glycolysis induced by BRAFi/MEKi treatment (220). Additionally, a large subset of melanomas is characterized by high levels of PGC1-α and increased OXPHOS metabolism independently of BRAF mutational status (81). Therefore, mitochondria and OXPHOS represent metabolic vulnerabilities to exploit for the design of new and more effective therapeutic strategies targeting both PGC1-α-positive melanomas and BRAF mutant melanomas (31). In BRAF-mutant melanomas, the use of drugs targeting mitochondrial biogenesis and mitochondrial metabolism in combination with BRAFi/MEKi may enhance and delay BRAFi/MEKi-induced cell death and resistance in melanoma, respectively (2). Furthermore, since mitochondrial addiction caused by MAPKi treatment makes melanoma cells more sensitive to mitochondria inhibition, the combination therapy with both oncogenic kinase inhibitors and mitochondrial inhibitors not only can increase MAPKi efficacy and delay resistance, but also can decrease doses of mitochondrial inhibitors and thus reduce toxicity to normal tissues (221). The co-treatment of melanoma cells with BRAFi and antidiabetic drugs phenformin and metformin, which strongly inhibit the complex I of the MRC, results in a synergistic inhibition of melanoma cell viability (Table 1) (222). Additionally, Yuan et al. also reported that phenformin can delay the appearance of BRAFi-resistant melanoma cells (222). Another potent and selective inhibitor of the complex I of MRC is represented by BAY 87-2243. This compound reduces the total cellular ATP levels, increases ROS production, and thereby leads to oxidative damage and subsequent cell death *in vitro*. BAY 87-2243 exerts a potent anti-tumor effect by reducing melanoma growth also in various mouse models *in vivo*. Interestingly, the inhibition of complex I caused by the use of BAY 87-2243 in association with vemurafenib (BRAFi) significantly reduces melanoma tumor growth *in vivo* when compared with their use as single agents (Table 1) (223). The efficacy of mitochondrial complex I inhibitors in melanoma treatment was also supported by Carpenter et al. who demonstrated that deguelin strongly suppresses the proliferation of vemurafenib-resistant melanoma cells by blocking the mitochondrial complex I. Of note, deguelin induces selectively toxicity only in cancer cells, without affecting the viability of normal human melanocytes *in vitro* (Table 1) (224).

**Table 1:** List of molecules targeting mitochondrial metabolism in CM.

**Agents**	**Mechanism**	**Metabolic target**	**References**
Phenformin/metformin	• Inhibition of complex I of MRC.	OXPHOS	(222)
BAY 87-2243	• Inhibition of complex I of MRC.	OXPHOS	(223)
Deguelin	• Inhibition of complex I of MRC.	OXPHOS	(224)
NSAIDs (diclofenac/lumiracoxib)	• Reduction of lactate release and MITF downregulation.	Glycolysis and OXPHOS	(157, 158)
SR4/ niclosamide	• Activation of AMPK, inhibition of mTOR and consequently induction of acute energetic stress.	OXPHOS	(219)
BAM15	• Inhibition of OXPHOS.	OXPHOS	(228)
Inhibitors of ERR	• Inhibition of ERRα.	OXPHOS and mitochondrial biogenesis	(229)
Inhibitors of TRAP1/TFAM	• Inhibitors of mitochondrial protein folding/mitochondrial genome replication and transcription.	Mitochondrial biogenesis	(232)
G-TPP	• Inhibition of TRAP1, involved in mitochondrial protein folding.	Mitochondrial biogenesis	(233)
PEITC	• Inhibition of glutathione S-transferase and complex I of MRC.	Glutathione metabolism	(234)
Sulfasalazine	• Inhibition of xCT.	Glutathione biosynthesis	(235)
Vorinostat	• Inhibition of histone deacetilase and suppression of SLC7A11 gene, which encodes xCT.	Glutathione biosynthesis	(239)
BPTES	• Inhibition of glutaminolysis.	Glutamine metabolism	(241)
BenSer	• Inhibition of the glutamine transporter ASCT2.	Glutamine metabolism	(242)
NAMPT inhibitors	• Reduction of NAD and ATP levels, depolarization of the inner mitochondrial membrane with loss of mitochondrial membrane potential and ROS release.	NAD biosynthetic pathway	(243)

Furthermore, it is known that NSAIDs, commonly used in clinical practice as cyclooxygenase inhibitors, induce cytotoxicity in various cancer cell lines, including melanoma cells (158). Additionally, Brummer et al. reported that two NSAIDs, diclofenac and lumiracoxib, reduce melanoma cell proliferation by targeting both respiration and glycolytic activity. In particular, diclofenac and lumiracoxib restrict energy metabolism by decreasing significantly both lactate release and OXPHOS via MITF down-regulation. Interestingly the combination of vemurafenib with either anti-metabolic NSAIDs is synergistic and results in a more pronounced decrease of proliferation and induction of cell death in human melanoma cells (Table 1) (157).

*In vitro* and *in vivo* studies revealed that SR4 and niclosamide, two small molecules mitochondria uncouplers, can be used successfully in the treatment of naïve wild type, BRAF^V600E^ and NRAS mutants, vemurafenib-resistant melanomas and melanomas with greater OXPHOS phenotype (Table 1). In fact, both SR4 and niclosamide, independently of BRAF or NRAS mutational status, promote energetic stress in melanoma cells by uncoupling mitochondrial OXPHOS, reducing intracellular ATP levels and consequently promoting the activation of the metabolic tumor suppressor AMP-activated kinase (AMPK) and the inhibition of mTOR pathway. As a result of this acute energetic stress, both the uncouplers promote cell cycle arrest and mitochondrial-dependent apoptosis in melanoma cells (219). Other studies supported the use of pharmacological direct AMPK activators, such as 5-aminoimidazole-4-carboxamide ribonucleoside (AICAR) (225, 226) and GSK621 (227), in the treatment of human melanomas. BAM15 is a second-generation mitochondrial uncoupling agent, which specifically inhibits OXPHOS with minimal off-target effects and consequences on cell viability. BAM15 causes cell death and alters proliferation when used in combination with low concentrations of PLX4032 or GSK1120212, which are BRAFi and MEKi respectively (Table 1) (228).

As discussed above, mitochondrial biogenesis is a drug resistance mechanism, activated by melanoma cells upon long-term exposure to MAPKi, and represents a promising therapeutic target in melanoma. PGC1-α, a key regulator of mitochondrial biogenesis and metabolism, is highly expressed in melanomas with OXPHOS phenotype and it is also upregulated in BRAFi/MEKi-resistant melanomas. However, the depletion of PGC1-α should be avoided in melanoma treatment because PGC1-α has a dual role in CM: it favors tumor growth and survival through the induction of oxidative metabolism and suppresses melanoma cell invasion and metastatic dissemination (229). Therefore, the depletion of PGC1-α in melanomas not only results in acute energy deficit caused by reduction of mitochondria metabolism (230), but also makes non-metastatic melanoma cells highly invasive (231). To overcome the dichotomy of PGC1-α, several studies were performed to identify components of PGC1-α protein complexes involved only in the metabolic regulation of mitochondria and not in the control of cell migration. Through these studies, the estrogen-related orphan nuclear receptors α (ERRα) was identified as a PGC1-α-dependent component, which promotes expression of genes involved only in OXPHOS, and not in the regulation of cell migration. Furthermore, *in vitro* and *in vivo* studies revealed that ERRα inhibition, as well as PGC1-α silencing, decreases mitochondrial respiration and cell proliferation in PGC1-α-positive melanoma cell lines. On the contrary, the inhibition of ERRα does not promote, unlike PGC1-α depletion, melanoma cell invasion. Taken together all these data suggest that ERRα may represent a promising metabolic target in the treatment of PGC1-α-positive melanomas (Table 1) (229). In addition to PGC1-α, other important regulators of mitochondrial biogenesis are represented by tumor necrosis factor receptor-associated protein 1 (TRAP1) and mitochondrial transcription factor A (TFAM). In particular, TRAP1 and TFAM are involved in mitochondrial protein folding and mitochondrial genome replication and transcription, respectively. However, Wu et al. reported that the inhibition of mitochondrial biogenesis through the depletion of TRAP1 or TFAM, but not of PGC1-α, increases the efficacy of MAPKi (Table 1) (232). Of note, gamitrinib, a small molecule inhibitor of TRAP1, synergizes with MAPKi and induces apoptotic cell death, mitochondrial dysfunction and suppression of tumor growth both *in vitro* and *in vivo* (Table 1) (80, 232). Furthermore, gamitrinib-triphenylphosphonium (G-TPP) was acquired through the attachment of a triphenylphosphonium (TPP^+^) group, which gives to gamitrinib a preferential tropism to mitochondria. Georg Karpel-Massler et al. demonstrated that G-TPP synergizes with inhibitors of anti-apoptotic Bcl-2 family proteins (BH3-mimetics) and together they increase intrinsic apoptotic cell death and reduce the tumor growth rate in orthotopic melanoma model. Interestingly, in BRAFi-resistant melanoma models, this combination therapeutic strategy safely and significantly prolongs host survival (233).

In MAPKi-resistant melanoma cells, the metabolic reprogramming toward mitochondrial respiration, leads to increased level of oxidative stress and ROS production. Consequently, in order to resist ROS generation and therefore to survive under oxidative stress, resistant melanoma cells enhance the metabolism of the antioxidant glutathione. Hence, glutathione metabolism can represent another promising metabolic target in melanoma treatment. BRAFi-resistant melanoma cells are resensitized to vemurafenib when treated with phenethyl isothiocyanate (PEITC), an inhibitor of glutathione S-transferase (234, 235), which is involved in cellular detoxification through the conjugation of glutathione to a wide range of substrates (236). Furthermore, PEITC could increase the cytotoxicity of BRAFi by a rapid depletion of glutathione and by inhibiting the mitochondrial electron transport complex I (Table 1) (235, 237). Additionally, to inhibit glutathione metabolism and thereby increase BRAFi therapy, melanoma cells can be treated with inhibitors of the cystine/glutamate antiporter (xCT), which mediates the uptake of cystine, a precursor for glutathione biosynthesis (Table 1) (238). Khamari et al. reported that sulfasalazine, an inhibitor of xCT, approved for the treatment of inflammatory bowel disease, is also able to delay the growth of BRAFi-resistant melanoma *in vitro* (Table 1) (235). Vorinostat, an histone deacetylase inhibitor (HDACi), enhances the levels of ROS in both MAPKi-sensitive and resistant melanoma cells by suppressing the expression of the Solute Carrier Family 7 Member 11 (SLC7A11) gene, encoding xCT (Table 1) (239). However, this leads to apoptotic cell death only in drug-resistant melanoma cells. In fact, in patients with melanoma resistant to BRAFi/MEKi therapy, vorinostat induces cell death only in drug-resistant melanoma cells. These data provide important clinical evidence for the use of vorinostat to eliminate new drug-resistant cells. Taken together all this evidence leads Wang et al. to assume that the pulsatile treatment with vorinostat, followed by a switch back to BRAFi/MEKi treatment, can get longer PFS benefit for melanoma patients with respect to the intermittent MAPKi only regimen (239). Furthermore, melanoma intracellular ROS over-production, caused by MAPKi treatment, can represent another important metabolic vulnerability in melanoma. In fact, it is possible to exploit ROS over-production in melanoma cells by using ROS activated pro-drugs in combination with MAPKi (Table 1). In particular, Yuan et al. demonstrated that A100 that is a ROS activated amine-containing compound, in combination with dabrafenib significantly suppresses *in vitro* melanoma cell proliferation and three-dimensional (3D) matrigel growth. Furthermore, they showed that A100 is able to restore sensitivity to BRAFi in BRAFi-resistant melanoma cells. Therefore, these results suggested that the combination therapy with a ROS activated pro-drug, such as A100, and a MAPKi, such as dabrafenib, could represent a potential strategy to treat BRAF-mutant melanoma patients and to overcome drug resistance (Table 1) (240).

MAPKi-resistant melanoma cells switch from glucose to mitochondrial glutamine metabolism and acquire the glutamine-addicted phenotype that represents a promising anti-tumor target. In fact, the use of the glutaminolysis inhibitor Bis-2-(5-phenylacetamido-1,2,4-thiadiazol-2-yl)ethyl sulfide (BPTES), in combination with BRAFi, enhances significantly the anti-tumor activity of BRAFi (Table 1) (241). Furthermore, glutamine transporter ASCT2 represents a potential therapeutic target for both BRAF wild type and BRAF^V600E^ melanoma. In fact, the knockdown of ASCT2 or its pharmacological inhibition by Benzylserine (BenSer) suppresses melanoma cell growth and proliferation (Table 1) (242).

Audrito et al. showed that when melanoma cells acquire BRAFi-resistance, NAD levels increase and the NAD biosynthetic pathway, relying predominantly on the rate-limiting enzyme nicotinamide phosphoribosyltransferase (NAMPT) becomes the dominant one. NAMPT that is involved in the conversion of nicotinamide to NAD, has been found upregulated in tissue biopsies from melanoma patients after the development of BRAFi resistance (242). Additionally, melanoma cell lines overexpressing NAMPT develop soon BRAFi resistance and grow rapidly. This makes NAMPT an actionable target for melanoma treatment. In fact, *in vitro* studies revealed that melanoma treatment with NAMPT inhibitors (NAMPTi) reduces NAD and ATP, depolarizes the inner mitochondrial membrane with loss of mitochondrial membrane potential, triggers ROS release, halts cells in the G2/M phase and induces apoptosis. Furthermore, NAMPTi reduce tumor growth and enhance survival in mouse xenograft models (Table 1) (243).

List of molecules, described in the text, and whose use in combination with MAPKi may overcome drug resistance and/or improve the effectiveness of current therapeutic strategies targeting melanoma, is showed in Table 1.

## Conclusions

CM tissue represents a very heterogeneous metabolic network influenced by metabolic reprogramming and plasticity of cancer cells whose interaction with TME leads to tumor progression and dissemination. The metabolic reprogramming of CM can be driven principally by BRAF^V600E^ mutant kinase, that induces upregulation of glycolysis also in normoxic conditions. BRAF^V600E^ mutation, glycolysis upregulation, structure and physiology of tumor vessels contribute dramatically to microenvironment acidification, which selects cancer cells with growth and proliferation advantages compared with non-transformed cells (2).

The capability of melanoma cells to shift their metabolic status between cytosolic glycolysis and mitochondrial respiration, in order to generate ATP and building block intermediates for cell growth, proliferation and dissemination, enables CM to modify dramatically its energetic pathways, in response to both microenvironmental changes and therapeutic strategies. In particular, the acidosis as well as BRAFi/MEKi treatment may switch the glycolytic metabolism of melanoma cells toward OXPHOS. The upregulation of mitochondrial metabolism correlates with increase in ROS levels and antioxidant systems and allows melanoma cells to offset glycolysis inhibition triggered by BRAFi/MEKi treatment. This process makes melanoma cells more dependent on mitochondria metabolism for proliferation and represents a weak point of cells resistant to MAPK inhibition. The integrated therapy with BRAFi/MEKi and molecules directed specifically toward cancer cell mitochondrial biogenesis and metabolism, and/or cellular antioxidant activity, is a promising strategy to counteract melanoma progression. In this complicated scenario, the microenvironment acidification, the interactions and the consequent metabolic crosstalk between melanoma and stromal cells sustain melanoma growth, and, by generating an immunosuppressive TME, can hinder immune response.

Therefore, a better elucidation of the melanoma metabolic alterations and crosstalk between cancer cells and TME may highlight further metabolic weakness and improve the efficacy of currently used therapies.

## Author Contributions

AAv and AAr planned the manuscript. AAv, AAr, GF, and AP wrote the manuscript. GS screened international scientific literature. MR and SM revised the manuscript and all of the authors accepted the final version.

## Conflict of Interest

The authors declare that the research was conducted in the absence of any commercial or financial relationships that could be construed as a potential conflict of interest.
